# Development of a New Knee Arthroscopy Operative Proforma Saving Thousands of British Pounds

**DOI:** 10.7759/cureus.48476

**Published:** 2023-11-07

**Authors:** Zain Habib, Sanjay Jain, Muhammad U Rasool, Sayantan Saha

**Affiliations:** 1 Trauma and Orthopaedics, Manchester University NHS Foundation Trust, Manchester, GBR; 2 Orthopaedics and Trauma, North Manchester General Hospital, Manchester, GBR; 3 Orthopaedics, North Manchester General Hospital, Manchester, GBR

**Keywords:** arthroscopic surgery, orthopaedics, medical records, arthroscopy, clinical coding

## Abstract

Coding inaccuracies in documentation of surgical procedures misrepresent the productivity of departments, with harmful fiscal consequences and detract from effective clinical governance. We aimed to assess the extent of this within our centres.

We retrospectively analysed the operative records of 34 patients from two centres over a period of a month, undergoing varying arthroscopic knee operations. We found that 50% of cases had incorrect coding for procedures performed. On review of the clinical coding, the loss of payment summed up to £29,325.

The flawed coding practices stemmed from the heterogeneity and convolution in documentation of procedures. Our intervention was the development of a multi-faceted arthroscopic operation note proforma, centred on concise documentation for appropriate codes to be gleaned.

We re-audited our new proforma, retrospectively collating data on 37 patients over a period of five months undergoing arthroscopic knee procedures. We found only 5% of cases were coded incorrectly, summing to a loss in tariff payment of £2654.

In conclusion, poor quality of documentation and written communication between surgical and coding departments can have drastic ramifications for funding. An active refinement of this process can ultimately help to provide more resources for improved patient care.

## Introduction

The national tariff system provides guidance for commissioners in assigning and governing funding for trusts based on the attested provision of services by secondary health providers. Prior to this Payment by Results system, assigned annual contracts were drawn up, dictating the clinical activity of a department in hospitals, and thus the overall funding [1]. The new tariff-based system introduced in 2004 aims to accelerate the productivity of services offered with the appeal of financial incentives for health boards. Clinical and operative coding is instrumental in depicting the modalities of how this care is provided, for trusts to be appropriately reimbursed [2]. Grouped interventions and diagnoses are converted into codes which utilise a similar amount of healthcare resources [3]. Operatively, this has been stratified by the use of Office of Population and Census Survey (OPCS-4) codes, a validated log of procedures with matching codes performed in the NHS [4]. Moreover, tariffs for procedures are also calculated by patient-specific data, such as co-morbidities and length of hospital admission [5].

Healthcare systems around the developed world have adopted this Payment by Results system, centred on the use of clinical codes to gage the productivity of healthcare provision [6]. The financial backing for healthcare, internationally, varies throughout coveted healthcare systems, sustained by either private or government funds. Nevertheless, appropriate reimbursement of services, pivots on the accuracy of clinical coding and its implementation by clinicians and practitioners alike [7].

There have been numerous documented reports of healthcare providers missing out on revenue due to the lacklustre coding of procedures [8]. Conversely, there have been reports of American health insurance companies combatting the embellishing effect of ‘over-coding’ [9].

The financial implications of inaccurate coding are not the only issues to consider. Coding provides an overview of patient care, establishing benchmarks for the propagation of future healthcare policy planning and effective clinical governance [10]. Coding provides records of quality of care delivered to patients, forming the crux of the evidence for national league ranking tables in terms of outcomes for individual practitioners [11].

Once documentation is completed by clinicians, it is the duty of non-medically trained clinical coders to transfer this into the assorted codes based on the frameworks aforementioned. It becomes an arduous task for clinical coders to accurately translate this documentation into codes if it is plagued by ambiguity and abbreviations. Illegible documentation is often either not transferred to a code at all or assigned the incorrect coding [12].

This issue is magnified when patients are undergoing specialised procedures; patients may have fine differences in diagnoses, indications and actual interventions performed under the same listed procedure.

The primary aim of this study was to assess the efficacy of introducing a novel coding-friendly proforma for practitioners performing arthroscopic knee procedures, on the subsequent accuracy of coding. The secondary aim was to evaluate the fiscal implications, following the implementation of the new orthopaedic arthroscopic knee procedure proforma.

## Materials and methods

Primary audit

Patients were included from two centres covered by the same trauma and orthopaedic service. We retrospectively collated data on patients undergoing knee arthroscopic operations over a period of five weeks, with a review of the medical records of each case. Each operative case under review was clinically coded blindly by authors, with details of the operator and patient anonymised. This was carried out by one author redacting medical notes with another author clinically coding each case based on the details of the operative note, basing our analysis on the ICD-10 and OPCS-4 codes provided by the coding department. Once this was done, the clinical codes that were originally assigned by coding staff were collated for each case. This data was subsequently compared to the coding data collated by the authors reviewing the redacted medical and orthopaedic operative notes. This was done to elicit any mistakes in the registered codes by the clinical coding staff. In liaising with the coding staff, we were able to devise an improved orthopaedic operative proforma, designed to avoid the pitfalls that could result in flawed coding of arthroscopic knee procedures and their outcomes.

Proforma development

The composition of the new proforma is centred on clear documentation in a structured fashion for arthroscopic procedures of the knee. The proforma provides space for hand-written operative diagnosis and procedures performed, as well as ample variety in tick box selections of common and uncommon arthroscopic knee operations. Its structure follows the approach in steps of an arthroscopy of a knee including ports used, with the initial section detailing the examination under anaesthesia. The following items are grouped in terms of their anatomical sections. The proforma includes salient features as per the Royal College guidelines for operative notes [13]. The detailed documentation this proforma encourages will have benefits in terms of audit and quality improvement projects. The layout and focus of tick box documentation would also allow this new proforma to function as a blueprint for the future digitalisation of operative notes for the related procedures performed in arthroscopy of the knee. 

**Figure 1:**
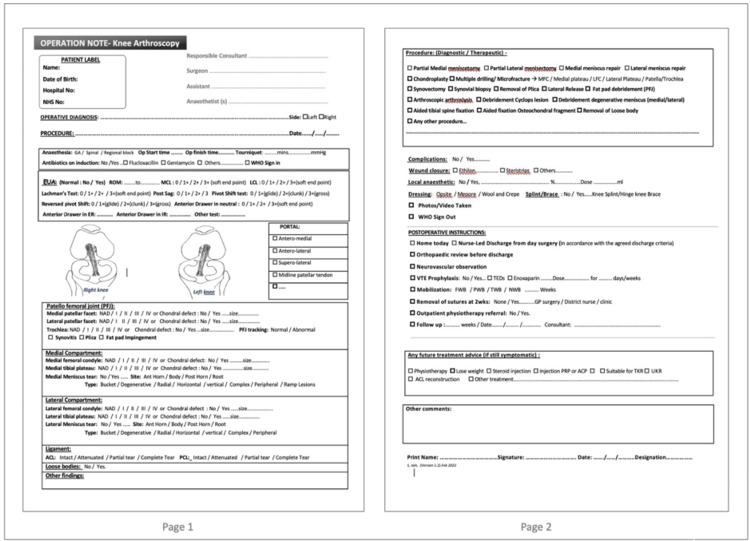
New proforma design

Once a blueprint for the new proforma had been established (Figure 1), this was introduced to the department in audit and clinical governance meetings. Feedback was collated on the proforma by senior practitioners with multiple open discussions with authors of the study present to ascertain if there were any adjustments to be made. The proforma was also discussed with nursing staff at opportunities such as day case morning handover meetings. The aim of this was for the nursing staff to become familiar with the new proforma before it was more widely implemented. Opportunities for feedback and alteration to the proforma were attained from clinicians at regular clinical governance meetings. This was done informally with authors taking notes on any perceived additional barriers to the uptake of the proforma from relevant clinicians. The final version of the proforma was established at a consensus meeting which included relevant stakeholders including consultants, senior nursing and coding staff. The document was disseminated throughout the department over a six-month period, via email and notice boards in clinical areas such as day cases, theatres and recovery. 

Re-audit

Consecutive data was collected over a period of five months on patients undergoing arthroscopic knee procedures, where the new orthopaedic proforma had been utilised to document their interventions. The protracted time period was required to accumulate the same number of patients in the data that was collated prior to the implementation of the new proforma. This was due to efforts in ensuring consistency in both legs of the audits pre- and post-production of the new proforma. We ensured proformas were only included that had been completed by practitioners that were included in the first audit. We also felt it was prudent to ensure the same clinical coding staff were involved. This ensured consistency pre- and post-intervention, adding to the reliability of the study. The re-audit cycle excluded data from any other coding staff, who were identified by their user log-in. This meant some cases had to be excluded from the second round of the audit; pre-empting this we purposely collected data on a few extra patients in the re-audit There was no evidence any of the coding staff were prone to errors than others, with mistakes in clinical coding being evenly distributed, each tallying at least three coding errors each. We included patients under the care of 11 different consultants who had received a knee arthroscopy procedure, reviewing the documentation via the new proforma and the assigned clinical codes through the data provided by the same cohort of coding staff for each corresponding case. 

A financial analysis was completed to understand the disparity in the monetary reimbursement from the clinical coding derived before and after the implementation of the new operative proforma. 

## Results

Primary audit

Thirty-four procedures were reviewed between April 2019 and May 2019 during the primary audit prior to the introduction of the new proforma. Analysis revealed that 50% of cases (n= 17) had been assigned an incorrect clinical code for the procedure performed. Based on the 2020-2021 tariff system, the monetary value of the clinical coding assigned to cases reviewed, summed to £85, 026. Once corrections were made to the coding, with the help of coding staff, this value came to £114, 351. This indicated a loss of value of £29, 325 (Table 1).

**Table 1:** Evaluation of coding during primary audit HRG: Healthcare Resource Group Codes

HRG Codes	Pre Audit	Post Audit	Difference
HN23B	2	5	
HN23C	5	17	
HN24B	3	0	
HN24C	20	8	
HN24E	1	1	
HN22E	2	2	
HT24D	1	1	
Total	£85,026	£114,351	£29,325

Re-audit

Thirty-seven procedures were collated following the implementation of the new operative proforma, between March 2022 and August 2022. Analysis depicted only 5% of cases (n=2) had been coded incorrectly. The remainder of the thirty-seven cases were coded correctly. As per the most up-to-date tariff, before and after the minor corrections that were made, the calculated reimbursement was £86, 886 and £89, 540 respectively. This indicated a relatively minor loss of £2654, in comparison to the period analysed prior to the implementation of the new operative proforma. Table 2 portrays further details in terms of the slight differences in the clinical coding once the data was audited for accuracy. Table 3 depicts a summary of the results from both legs of the audit. 

**Table 2:** Evaluation of coding during re-audit following implementation of new proforma HRG: Healthcare Resource Group Codes

HRG	Pre-audit	Post-audit	Difference
HN23B	1	1	
HN23C	8	9	
HN24B	1	1	
HN24C	25	25	
HN24E	1	1	
HN25A	1	0	
Total	£86, 886	£89, 540	£2, 654

**Table 3:** Summary of results

Primary Audit Pre-New Proforma	Re-Audit Following Implementation of a New Proforma
Out of 34 cases 17 coded incorrectly (50% coded incorrectly)	Out of 37 cases 2 coded incorrectly (5% coded incorrectly)
Calculated total before corrections £85,026	Calculated total before corrections £86, 886
After Corrections £114,351	After Corrections £89,540
Difference £29,325	Difference £2654

## Discussion

Inaccuracies in clinical coding have been documented to be a common problem for many centres around the world. This is not an issue which is unique to our department of orthopaedics and has been noted to be ubiquitous in various specialities. Data from the comprehensive 2013/2014 audit into clinical coding across NHS trusts showed inaccuracy rates as high as 45%, comparable to the 50% rate in our department, prior to the introduction of the new coding-friendly operative proforma by the authors [14]. It seems counterintuitive that clinicians would turn a blind eye to a key systematic financial flaw, in ultimately ensuring the delivery of the best possible care to patients. This potentially can be caused by a fundamental lack of awareness of the importance of clinical coding and its direct effect on the outcome of the resources available for patients. It is understandable that clinicians maintain their focus on the point of care for patients; however, it is a pertinent governance issue to ensure all healthcare providers are informed on how poor clinical coding can undermine the quality of a healthcare service [15].

As this study showed, there is a lack of awareness among surgeons regarding the details of how certain procedures should be documented for accurate translation into clinical codes. Often surgeons will not document the full extent of the pre-op diagnoses or planned procedure. This includes not detailing diagnoses from intra-operative findings, with partial fleeting documentation of additional procedures performed directly as a result of them [16]. The most common cause of inaccurate coding from our data was vague documentation of preoperative diagnoses, with the absence of clear documentation of additional procedures performed. For example, a patient had a pre-op diagnoses of a medial meniscus tear. Intraoperative arthroscopic findings revealed an additional degenerative lateral meniscus tear, which required a further lateral partial meniscectomy. However, this case was only coded for a partial medial meniscectomy (code HN24C), whereas it should have been coded as a bilateral partial meniscectomy (code HN23C). Moreover, grade 3 cartilage changes were seen throughout the knee which required debridement, which were not accurately coded for. Cartilage changes causing unexpected further operative burden were a theme running throughout arthroscopic coding inaccuracies noted during the audit.

Despite the complexity of various arthroscopic knee procedures, these are often completed as day cases, resulting in a high workload for operating surgeons. Due to the high turnover of patients, this might result in suboptimal documentation of procedures, adding to inaccuracies in clinical coding. A systematic review looking into coding in England and Wales showed significant inaccuracies, of up to 20% more in documentation of operative procedures, as opposed to other aspects of data such as clinical coding for diagnoses [17]. A high turnover of patients on a day case arthroscopy list can also add to the issue of poor clinical coding generated from discharge summaries, as clinicians are not provided with enough time for this to be as comprehensive as possible. Discharge summaries are often delegated to junior members of staff including foundation year doctors, who are not always able to extensively and accurately document procedures and intra-operative diagnoses [18]. Accuracy in coding data synthesised from discharge summaries, as well as operative documentation, is a salient issue that must be addressed; in the UK clinical coding from discharge summaries forms the basis of assessing the productivity and performance of NHS trusts and hospitals and can often be the only information clinical coding staff have access to [18]. 

The novel orthopaedic proforma relies on tick box documentation, along with a layout based on the anatomy encountered in a knee arthroscopy; offering a blueprint for which the proforma can easily be converted into a digital format. Studies have shown that handwritten notes are a detriment to the accuracy of clinical coding, as opposed to notes that have been digitalised and typed up [19]. Tick box documentation, in essence, allows clinical coding to be completed by the operating surgeon at the time of the procedure, making the job of clinical coders rather administrative instead of investigative. In the future, it is viable to envisage clinical coding itself to also be digitalised, as hospitals move towards computerised medical records. This would dramatically decrease the resistance to representative coding of clinical activity, decreasing the administrative burden it currently holds. Studies have demonstrated that a higher standard of medical documentation leads to improved clinical and diagnostic coding [20]. However, clinical coding staff are able to review operation notes before assorting operative codes and have an investigative responsibility to ensure these codes are correct. In searching for a solution for this issue, practitioners and surgeons should not become bogged down in repetitive documentation, as this can detract away from effective patient care.

The payment by tariff system was brought in to instigate an increase in the productivity of healthcare trusts incentivised by financial reward. However, it is important to note the payments by tariff system introduces an administrative burden itself. It is estimated the administrative cost of the tariff system to each trust is around £180, 000 a year [21]. Our study demonstrates the potential to improve the efficiencies of the current system at our trust. 

Certain surgical specialties seem to be more prone to others in being susceptible to suffering from clinical coding errors. This can be explained by the cohort of patients being treated, the complex and variable nature of procedures and subtle differences in procedures causing them to be bundled under incorrect umbrella codes. The subjective and heterogenous nature of the clinical coding of arthroscopic and otolaryngology procedures causes these sub-specialities to face similar barriers to the efficiency of the pay by tariff system. A large study in 2013 looking into clinical coding of ENT procedures found that almost 25% of interventions had been designated inaccurate clinical codes, causing a significant detriment to the fiscal reimbursement based on the centre's tariff system [11].

Specialisation in a certain aspect of the medical field is the norm for clinicians and healthcare staff alike, and proposing for this to be the structure adapted by clinical coders might allow the process to be more successful. This is not to suggest that the responsibility for effective coding should completely be devolved to clinical coding staff.

There are some limitations to this study. The sample size for both legs of the study, prior and post-implementation of the new operative proforma, were small; with only 71 patients in total being analysed. The extended period of five months taken for the second leg of the study was on account of the slow improvement in the compliance of the new proforma and consequently, some data were excluded due to incorrect use of the form. In addition to this, the short 5-week period opted for in the first leg of data collection can also be criticised, as this is not congruent with the timeframe allotted to the reaudit. More time was also allowed for the second leg of data collection due to the delay in the implementation and uptake of the new proforma despite our best efforts. The authors of the study aimed to gather a snapshot of the clinical coding ascertained from operative documentation. There was no evidence any of the coding staff were prone to errors than others, with mistakes in clinical coding being evenly distributed, each tallying at least three coding errors. The numbers in both data sets were similar and the authors believe the study conclusions are valid despite these minor limitations. 

In our centre, knee surgeons typically perform between 80 and 90 arthroscopies each year. Reviewing the data for one of the knee surgeons, they performed on average 89.2 arthroscopies per annum over the last five years. Extrapolating the findings of this study, this might mean an increased income of £76,935 for the centre per surgeon yearly, based on these figures of incorrect clinical coding which can be addressed successfully, as depicted by this project. 

## Conclusions

Coding inaccuracies in documentation of surgical procedures misrepresent the productivity of departments, with harmful fiscal consequences and detract from effective clinical governance. This study demonstrates the significant financial setback for centres that can stem from inaccurate coding. Streamlining the documentation process in order to improve written communication between clinicians and clinical coding staff has been shown by this study to be an effective strategy to combat the root cause of this. A greater emphasis should be placed on clinicians to develop methods alongside coding departments to improve the process and accuracy of clinical coding.
